# Analysis of Clinical Target Volume Delineation in Local-regional Failure of Nasopharyngeal Carcinoma after Intensity-modulated Radiotherapy

**DOI:** 10.7150/jca.39588

**Published:** 2020-01-29

**Authors:** Xiaojing Yang, Hanru Ren, Weiwei Yu, Xiulong Zhang, Yi Sun, Yuhui Shao, Lihua Zhang, Hongling Li, Xinmiao Yang, Jie Fu

**Affiliations:** 1 Department of Radiation Oncology, Shanghai Jiao Tong University Affiliated Sixth People's Hospital, No. 600, Yishan Road, Shanghai, 200233, China.; 2 Department of Orthopedics, Shanghai Pudong Hospital, Fudan University, Pudong Medical Center, Shanghai 201300, P.R China

**Keywords:** Clinical target volume, Delineation, Nasopharyngeal carcinoma, Intensity-modulated radiotherapy

## Abstract

OBJECTIVE: To analyze the pattern of local failure in patients with nasopharyngeal carcinoma (NPC) after intensity-modulated radiotherapy (IMRT) and find a more reasonable delineation of the clinical target volume (CTV).

METHODS AND MATERIALS: A total of 212 patients with non-metastatic NPC who underwent IMRT were analyzed. Radiation therapy was run at a total dose of 66-74 Gy (2.0-2.2 Gy fractions). The follow-up of local recurrence and the recurrence-related features were analyzed for the original treatment situation. The failures were delimited as “in-field failure” if V_recur_ within the 95% isodose curve (V95%) was ≥95%; “marginal failure” if V95% was less than 95% and not less than 20%; or “out-field failure” if V95% was< 20%. Kaplan-Meier method was used to calculate the survival rates.

RESULTS: The median follow-up was 43.4 months. The 5-year local relapse-free survival and overall survival rates were 85.6 and 77.8%, respectively. A total of 18 patients have relapsed. The in-field failure, marginal failure, and out-field failure accounted for 83.3%, 11.1%, and 5.6%, respectively. The site of recurrence was basically in the high dose area.

CONCLUSION: These findings suggested that IMRT provide a good local control for patients with NPC, and the in-field failure is the main mode. A wide range of CTV cannot prevent the local recurrence, narrowing the CTV to protect the adjacent organs should be taken into consideration.

## Introduction

Nasopharyngeal carcinoma (NPC) is the most common cancer of all head and neck malignancies in Southeast Asia. Radiation therapy is regarded as the most effective treatment for NPC 1, and intensity-modulated radiotherapy (IMRT) has been widely used in radiation oncology. Over the past decade, IMRT has been considered to be a major breakthrough in the treatment of NPC due to its ability to offer high radiation doses to targets while preserving adjacent organs 2, 3.

However, local recurrence remains to be the leading cause of failure in NPC patients with locally advanced disease 4. Dawson et al. 5 in the year 2000 are the first to study the connection between recurrence and previous therapeutic dose distribution. After 27 months of follow-up, the results revealed that most of the local recurrence after IMRT treatment was an “in-field” recurrence, which included the gross tumor volume (GTV) and surgical boundary. In the previous years, there are several studies on the analogous failure patterns in NPC patients 4, 6-8. However, Lin et al. 9 have reported that the IMRT used has reduced the volume in NPC patients, and the results showed a similar local control. This suggested the possibility of lowering the clinical target volume (CTV) without affecting the local control rate. NPC patients usually have a longer life expectancy and are more prone to long-term complications due to radiotherapy 10. The complications such as hearing loss, cranial nerve damage, endocrine dysfunction, and temporal necrosis of temporal lobe that are caused by radiotherapy 11,12,13,14,15 can lead to decreased quality of life in NPC patients 16, 17. The most significant effort required to ameliorate the outcomes might be an accurate delineation of CTV, which has been a great challenge for radiation oncologists.

Hence, in our study, the initial dose and the local failure pattern of recurrence sites in NPC patients were studied in detailed to present the importance of CTV. The summary of our research revealed that CTV delineation could be further improved to enhance the life quality of NPC patients without reducing local control and long-term survival.

## Materials and methods

### Patient characteristics

A total of 212 patients with NPC who underwent IMRT in our department between September 2008 and December 2018 were retrospectively collected. These NPC patients were newly diagnosed, untreated, and had no distant organ metastasis. The detailed clinical data of patients were presented in Table 1.

### Diagnostic criteria

All cases received pretreatment assessments involving integral medical history, physical examination, hematology data, Magnetic Resonance Imaging (MRI) scans of the nasopharynx and neck, chest radiographs, and abdominal ultrasound. The retrospective analysis of medical and imaging records of all cases was done according to the American Joint Committee on Cancer (AJCC) version 6 staging system.

### Radiotherapy target volume definition

The ICRU Report Nos. 50 and 62 were referred to define the target. GTV was divided into (1) primary foci GTV (GTV-P) include primary nasopharyngeal tumors and posterior pharyngeal lymph nodes; and (2) lymph nodes GTV (GTV-N) include all cervical metastatic lymph nodes. CTV was divided into high-risk volume (CTV1), which included the high-risk CTV in the nasopharynx and neck. (1) The high-risk CTV of the nasopharynx includes the entire nasopharynx, posterior pharyngeal lymph node area, skull base, slope, parapharyngeal space, pterygopalatine fossa, sphenoid sinus, part of the nasal cavity and 1/3 of the maxillary sinus, and includes the complete GTV; (2) low-risk area CTV2 includes the lymph node area of the neck IV and Vb areas. There are 4 PTVs in this study: PTV-G (GTV + 5 mm), PTV-N (GTV-N + 5mm), PTV-C1 (CTV1 + 3 mm) and PTV-C2 (CTV2 + 3mm).

### Radiation treatment planning

All patients underwent IMRT for 6-MV photon external irradiation. The prescription doses and segmentation methods were as follows: PTV-G: dose was 66Gy in 30 fractions for stage T1 and T2; dose was 72-74 Gy in 32-33 fractions for stage T3 and T4; PTV-N: 66 Gy in 30~32 fractions; PTV-C1: 60 Gy in 30~32 fractions; PTV-C2: 54 Gy in 30~32 fractions.

### Chemotherapy

The majority, i.e., 92%, of patients underwent chemotherapy based on cisplatin, including induction, concurrent or adjuvant chemotherapy. The commonly used chemotherapy regimens include PF regimen (cisplatin 25 mg/ m^2^/day, for three days + fluorouracil 0.5 g/m^2^/day, for three days), TPF regimen (docetaxel 60 mg/m^2^/day, for first day + cisplatin 25 mg/ m^2^/day, for three days + fluorouracil 0.5 g/m^2^/day, for three days), and GP regimen (gemcitabine 1 g/m^2^/day, for first and eighth day + cisplatin 25 mg/m^2^/day, for three day). Concurrent chemotherapy was performed with cisplatin 80 mg/m^2^/day for three days, once every three weeks.

### Follow-up

Follow-up time was defined as day one from treatment till death or the last followed up time. Patients were followed up once a week during radiotherapy to assess tumor regression and normal tissue adverse events. After the end of the treatment, the patients were followed up every 3 months for the first 2 years, and every 6 months from 3-5 years. After that, follow-up was performed once a year subsequently. Follow-up included physical examination, MRI or computer tomography (CT) of the nasopharynx, chest CT, and abdominal ultrasound. Patients were subjected to cervical MRI or CT, electronic nasopharyngoscopy and emission computed tomography (ECT) for bones if necessary. Imaging examinations were done 3 months after the end of radiotherapy and were performed every 6 months. After 5 years, the follow-up was carried out once every 1 year.

### Definition of failure mode

The diagnostic criteria for recurrence included pathology, cytology, and imaging diagnosis. Patients with local or regional recurrence were screened, and MRI or CT was introduced for scanning the recurrence into the treatment planning system (TPS). The anatomical structure and the bony mark were used to fuse with pre-treatment CT, and the recurrence range (V_recur_) was delineated. The treatment plan for the first-pass radiotherapy was replicated and calculated according to the original prescription dose. The recurrent lesions were evaluated by dosimetry. Evaluation indicators included D_max_, D_min_, D_mean_, and V95% as received by V_recur_. The failure modes were defined as follows: V95% ≥ 95% is defined as recurrence in-field, 20% ≤ V95% <95% for marginal failure, and V95% < 20% for out-field failure 18.

### Statistical analysis

Kaplan-Meier analysis and log-rank tests using SPSS 23.0 (IBM Corporation, Armonk, NY) were used to present the survival data. Multivariate analysis was executed using the Cox risk regression model.* P* values of less than 0.05 were considered to be statistically significant.

## Results

### Patient characteristics and survival

**Figure 1:**
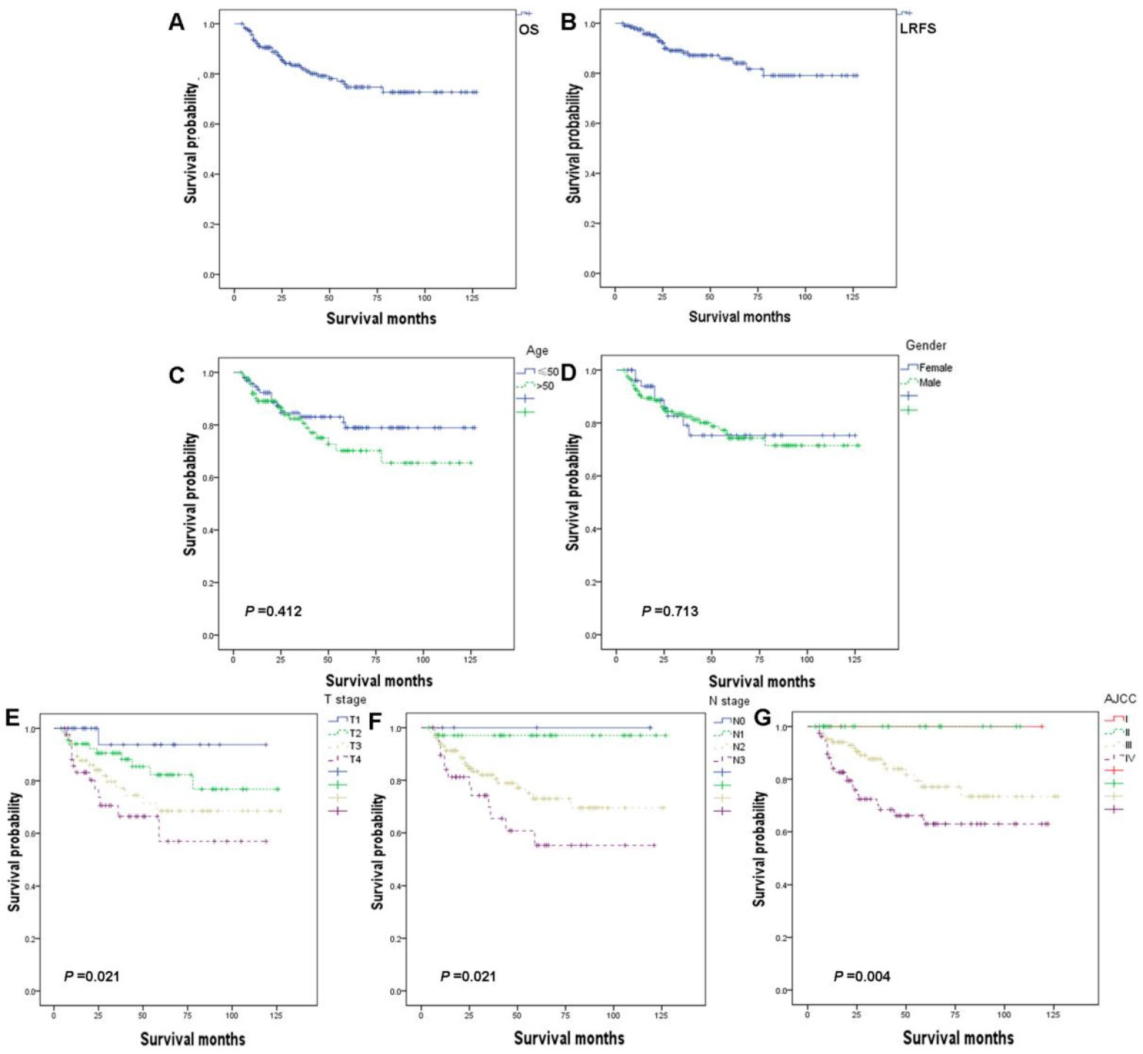
Kaplan-Meier analysis of overall survival (OS) (A) and local relapse-free survival (LRFS) (B) among patients, stratifed by age (C), gender (D), T stage (E), N stage (F), and AJCC stage (G).

A total of 212 NPC patients were analyzed. The epidemiological properties of the patients, tumor staging and treatments were shown in Table 1. There were 158 male patients and 54 female patients with a ratio of 2.9:1. The median age of the patients was 53.5 years (range 18-84 years). Histologically, most of the patients included had nonkeratinizing carcinoma, and only 4 patients had keratinizing squamous cell carcinoma. The overall staging of the patients was as follows: T1, 30 patients (14.1%); T2, 68 patients (32.1%) T3, 68 patients (32.1%); and T4, 46 patients (21.7%); N0, 5 patients (2.4%); N1, 38 patients (17.9%); N2, 130 patients (61.3%) and N3, 39 patients (18.4%); and stage I, 4 patients (1.9%); stage II, 27 patients (12.7%); stage III, 102 patients (48.1%); stage IV, 79 patients (37.3%). Follow-up time ranged from 4 months to 127 months, and the median follow-up of 34 months. The 5-year overall survival (OS) and local relapse-free survival (LRFS) were 77.8% and 85.6 %, respectively (Fig. 1A, 1B).

Age (Fig. 1C) and gender (Fig. 1D) showed no significant relationship with prognosis. The higher T stage (Fig. 1E), N stage (Fig. 1F), and AJCC stage (Fig. 1G) showed negative correlation to OS by univariate analysis model. Further, multivariate Cox regression analysis of these factors was conducted and the results showed no significant correlation (Table 2).

### Rates of local-regional recurrence

At the end of the follow-up period, localized recurrence occurred in 18 patients (8.5%). Of these, 94.4% (17/18) patients were locally advanced patients, and most of them are in stages III and IV. The median time from the first treatment to recurrence was 24.5 months (range 7 to 73 months). Of these cases with recurrence, 11 of them were well rescued only through surgery (2), chemotherapy (3), RT (1), cervical lymph node dissection and RT (2), or RT combined with chemotherapy (3). Seven patients had unrecoverable local area recurrence, in which 1 patient had distant metastases before, during or after local recurrence, while 2 patients have refused any treatment.

### Dosimetric analysis of patients with recurrence

The dose distribution of the target volumes in the first-pass radiotherapy of relapsed patients was shown in Table 3. Both GTV-P and GTV-N had higher dose coverage. Only 1.6% of the GTV-P and 0.5% of the GTV-N obtained <95% of prescription dose. A greater part (92.7% to 94.3%) of GTV-P and GTV-N obtained ≥100% of prescription dose. The doses of CTV1 and CTV2 used were similar. The mean doses of CTV1 and CTV2 were 64.3 and 55.7 Gy, respectively, and the <95% prescription dose volumes were 0.9% and 0.6%, respectively.

### Local and regional failure mode

Dosimetric analysis of recurrent lesions of these 18 patients showed that 83.3% of the recurrent lesions were in the 95% isodose curve and belonged to the in-field recurrence. Marginal failure and out-field failure accounted for 11.1% and 5.6%, respectively (Table 4).

As shown in Table 4, the average minimum dose for the V_recur_ in the in-field recurrences was 63.2Gy (range 58.5Gy to 67.4Gy). The average mean dose was 67.1Gy (range 63.1Gy to 71.2Gy), and the average maximum dose was 74.6Gy (range 69.4Gy to 78.6Gy) for the V_recur_ in the in-field recurrences.

## Discussion

IMRT has become the first choice for radiotherapy in patients with NPC. In recent years, various treatment centers have reported encouraging treatment results. Kwong et al. 19 have reported that 50 locally advanced patients had a local control rate of 95.7% after 2 years of IMRT; Wang et al. 20 have revealed that 300 NPC cases who underwent IMRT had LRFS and OS of 94% and 86%, respectively; and Lai et al. 21 have shown 5 years of LRFS and OS as 92.7% and 75.9%, respectively. Kong et al. 7 have studied the survival of 370 NPC cases treated with IMRT, and the 2-year OS was 94.1%. In our study, the 5-year LRFS and OS were 85.6 and 77.8%, respectively. Our results are similar to those reported in the above literatures, and long-term results need further observation.

Dosimetric analysis of recurrent lesions showed that the main recurrence pattern after IMRT treatment for patients with NPC was the in-field recurrence. With this, we hypothesized that most of the recurrences occur in high-dose areas, and so can the CTV range in some patients be reduced accordingly? This is because even in the large-scale and large doses of radiation, these recurrences cannot be avoided. Can we reduce the scope of CTV during the first radiotherapy to alleviate the side effects of radiotherapy in patients still remains a question. Salvage radiotherapy or surgery is performed again when relapsed in a small number of patients with recurrence. The radiotoxicity of NPC is very common and might be partly related to the non-standardized CTV. There are no predefined guidelines to direct the CTV and clinical outcomes. According to a study conducted by Sanford et al. 22, individualized CTV techniques were used to treat NPC patients and reduced the volume of CTV. Through long-term follow-up, high local control of the patients' lesions and low toxicity of normal organs were achieved. The treatment plan of this study included patients with nasopharyngeal lesions on only one side, and CTV only covers the ipsilateral parapharyngeal space to reduce the occurrence of closed jaw closure. CTV does not routinely include nasal, maxillary, sphenoid or ethmoid sinus. To reduce the dose to the mouth and soft palate, CTV does not cover the pharyngeal airway, reducing the incidence of mucositis or dry mouth. Combined with the data about the recurrence of our patients, individualized CTV was designed for patients who require radiotherapy in the future according to the actual situation of patients and CTV treatment range of Sanford et al., to minimize the side effects of radiotherapy.

Two patients in our study had a marginal recurrence. The first patient (Table 3, patient 5) had a relapse in the skull base and cavernous sinus, and had intracranial invasion before radiotherapy. The lowest dose of PTV-G was 57.4 Gy, and V_95%_ was 9.2%, suggesting that the patient had insufficient irradiation dose after first-time radiotherapy. In the second patient (patient 14 in Table 3), there was a recurrence of pterygopalatine fossa, and the patient had extensive skull base invasion before radiotherapy. The lowest dose of PTV-G was 65.8 Gy and V_95%_ was 9.3% during the first-pass radiotherapy. We considered that the patient's recurrence was associated with the local low dose. The formation of local low-dose areas in the above two patients might be due to dose limitation of the optic nerve and optic chiasm. For patients with locally advanced, especially in stage T4, the dose of radiation therapy has become a problem in clinical treatment. The dose limitation has become an important factor for organ at risk (OAR), such as optic chiasm, optic nerve, brain stem, and so on, and so the local doses are difficult to reach the radical dose, even under the conditions of IMRT 23. Some researchers have suggested that dose limitation of normal tissue (such as sacrificing the side of the optic nerve or temporal lobe) can be selectively reduced to achieve the goal by increasing the dose at the tumor target. However, whether the benefit of this method for patients with NPC is greater than the effect on quality of life still remains debatable.

The determination of the extent of CTV based on primary lesions plays a vital role in the control of local tumors as well as in the protection of normal tissues. The current definition of CTV is mostly based on the experience with 3 dimensional or conventional radiation fields, which had a lack of individualization and different dose gradients. Even though the Radiation Therapy Oncology Group (RTOG) 0225 and 0615 18 provided a reference for the CTV-1 range of NPC, the best definition of primary CTV has not yet been defined. In 2009, Liang et al. 24 have analyzed data from 943 NPC patients who underwent nasopharynx and neck MRI. After review by two radiologists, a low risk of bilateral tumors in the nasopharyngeal area (10%) was detected. Local lesions gradually spread from the proximal part to the more distal part, while the local extension in a jumping pattern is uncommon. Thence, when the tumor erupts on the side of the nasopharynx, the high-risk bilateral anatomy should be covered by the CTV, while the moderate or low hazard and the contra lateral site ought to be ruled out from the CTV. Lin et al. 25 have analyzed data from 414 NPC patients who underwent IMRT treatment and defined CTV by GTV expansion of 5-10 mm margins in various paths, which included the whole nasopharyngeal mucosa and 5 mm submucosal volume. After follow-up for 5-years, the local recurrence showed no increase in the marginal correlation that is associated with the method used. For T4 disease, the flared edges of the six directions of the mass were notably smaller than those of the entire patient group. Hence, CTV-1, including GTV expansion of 5-10 mm, the whole nasopharyngeal mucosa and a 5 mm sub mucosal volume was established. These results indicated that the target volume applied by the Fujian Medical University Cancer Hospital is sufficient. On the other hand, our organization used symmetrical coverage depicted by CTV and achieved good local control. By analyzing the location of the recurrence site, a guide to the CTV target volume was presented. According to our study, most of the anatomical sites are associated with low risk of bilateral tumors, and most of the local recurrence sites are located at the same site. We recommended that the contra lateral side of the tumor zone could be ruled out from the CTV.

CTV reduction can reduce the range of exposure to the contra lateral and mid-lower neck of the lesion, providing better protection to the OAR located in the area. Currently, even with IMRT, neck OAR might be subjected to varying degrees of damage. Acute and advanced dysphagia is a common complication after radiation therapy, with a total incidence of 18%-93.5% 26, 27. Symptoms of advanced dysphagia are associated with patients for many years 26, affecting the quality of life of the patients. The RTOG 0615 guidelines recommend an average dose of no more than 45 Gy for these OARs. Tiziana and his colleagues suggested that lessening the bulk of pharyngeal contractile muscles and larynx assists in receiving radiation doses of ≥ 60 Gy. Further reduction of the volume of ≥ 50Gy when possible is related to dysphagia and aspiration 28. Similarly, radiation usually causes hypothyroidism (HT) and its incidence is 20.50% 29, 30. If the thyroid gland receives radiation therapy dose of more than 45Gy, the occurrence of HT shows a significant increase 31. Reducing CTV in the contra lateral neck region definitely reduces the dose of pharyngeal contractile muscles, esophagus, larynx, and carotid artery.

As displayed in Table 3, high local recurrences appeared in the high dose area. In our study, there was only one external field recurrence. The No.15 patient's stage was T2N2M0, and was accompanied by parapharyngeal space invasion. The first treatment included radiotherapy combined with concurrent chemotherapy, but the patient showed recurrence at 28 months after initial treatment. A retrospective study of pre-treatment MRI did not reveal any ethmoidal sinus disease. He survived till date (44 months) after receiving salvage chemotherapy and IMRT. Ng et al. 4 informed that one case had failure after one year of treatment, and the recurrence site was mainly located in the maxillary sinus and ethmoid sinus. The patient's stage was T3N2 without ethmoid sinus invasion before treatment. Is this likely to indicate another primary tumor? As the ethmoid sinus is a very rare site of NPC recurrence, it requires large number of samples and longer follow-up to determine if the posterior ethmoid ought to cover in high risk CTV.

However, our research has some limitations. Firstly, this study was a retrospective analysis with some bias. In the future, it is necessary to conduct randomized controlled clinical trials. Secondly, due to various factors such as time and location of the tumor, 100% accuracy of image fusion and dose analysis cannot be guaranteed. Thirdly, patients enrolled in this study underwent different chemotherapy regimens, showing varied impacts on the prognosis. Fourthly, this is a single-center data analysis, and multi-center clinical trials should be conducted in future to obtain more comprehensive data.

Our study analyzed local failure patterns after IMRT treatment in NPC patients. Recurrence in this field of research is the main mode of local recurrence, and a wide range of high doses of radiation cannot prevent local recurrence. Most of the local recurrence sites are located in the same site of the primary tumor, while bilateral tumors rarely occur. Therefore, we proposed that the range of the target volume of patients with NPC receiving IMRT treatment can be appropriately reduced.

## Figures and Tables

**Table 1:** Patient characteristics

Subject characteristics	Number of patients	%
**Age**		
≤50	94	44.3
>50	118	55.7
**Gender**		
Female	54	25.5
Male	158	74.5
**Histologic type**		
Keratinizing squamous cell carcinoma	4	1.9
Nonkeratinizing carcinoma	208	98.1
**T stage**		
T1	30	14.1
T2	68	32.1
T3	68	32.1
T4	46	21.7
**N stage**		
N0	5	2.4
N1	38	17.9
N2	130	61.3
N3	39	18.4
**AJCC stage**		
I	4	1.9
II	27	12.7
III	102	48.1
IV	79	37.3
**Radiotherapy**		
Median dose (Gy)	70	
Range (Gy)	66-74	
**Chemotherapy**		
No	17	8.0
Induction	12	5.7
Concurrent	11	5.2
Induction+ Concurrent	34	16.0
Concurrent+adjuvant	51	24.1
Induction+ adjuvant	24	11.3
Induction+ Concurrent+ adjuvant	63	29.7

**Table 2:** Multivariable Cox regression for analyzing the prognosis factors for NPC

Subject characteristics	No. of NPC patients		HR (95%CI)	*P*
	overall	Dead (N, %)		
Age				
≤50	94	16(17.0)	1 (Reference)	1.0
>50	118	24 (20.3)	1.462 (0.775-2.761)	0.241
Gender				
Female	54	9 (16.7)	1 (Reference)	1.0
Male	158	31 (19.6)	1.110 (0.528-2.332)	0.783
T stage				
T1	30	1 (3.3)	1 (Reference)	1.0
T2	68	10 (14.7)	3.783 (0.484-29.563)	0.205
T3	68	16 (23.5)	6.583 (0.873-49.650)	0.683
T4	46	13 (28.3)	1.270 (0.194-74.403)	0.028
N stage				
N0	5	0 (0.0)	1 (Reference)	1.0
N1	38	1 (2.6)	1260.365 (0.000-1.301E83)	0.939
N2	130	26(20.0)	10518.969 (0.000-1.075E84)	0.922
N3	39	13 (33.3)	18317.667 (0.000-1.873E84)	0.917
AJCC stage				
I	4	0 (0.0)	1 (Reference)	1.0
II	27	0 (0.0)	1.001 (0.000-1.021E62)	1.0
III	102	17 (16.7)	2929.344 (0.000-8442E60)	0.906
IV	79	23 (29.1)	5885.555 (0.000-1.696E61)	0.898

Abbreviations: NPC, nasopharyngeal carcinoma; IQR, interquartile range.

**Table 3:** Dose-volume histograms (DVHs) statistics for patients of local-regional recurrence

	GTV-P	GTV-N	CTV1	CTV2
	Average (range)	Average (range)	Average (range)	Average (range)
Dmax	76.3 (72.1-78.4)	73.7 (71.7-76.0)	74.8 (69.9-78.2)	62. (59.3-64.1)
Dmean	72.3(67.5-73.5)	68.6 (67.5-70.6)	64.3 (61.6-70.2)	55.7 (53.2-58.1)
Dmin	57.3 (50.8-64.4)	57.1 (50.1-65.3)	42.9 (22.6-58.5)	40.7 (15.8-55.3)
V95%	1.6 (0-5.7)	0.5 (0-1.9)	0.9 (0-1.8)	0.6 (0-2.0)
V100%	92.7 (79.3-98.5)	94.3 (91.3-99.0)	95.8(93.0-99.2)	95.9 (92.4-98.9)
V110%	0.2 (0-0.9)	0.6 (0-2.6)	0.9 (0-3.7)	2.2 (0-5.6)

GTV-P gross tumor volume of primary tumor, GTV-N gross tumor volume of involved lymph nodes, CTV1 clinical tumor volume of the high-risk region, CTV2 clinical tumor volume of lymph nodal regions at low risk. Dmax = Maximum dose, Dmean = Mean dose, Dmin = Minimum dose. V95% =%volume receiving < 95% of the prescribed dose, V100% =% volume receiving >100% of the prescribed dose, V110% =% volume receiving >110% of the prescribed dose.

**Table 4:** Details of local -regional recurrence patients

No. of Patient	Age (years)	Sex	Stage	Site of relapse	Location of the recurrence volume	DVH statistics to recurrence volume				Type of relapse
						Dmean (Gy)	Dmin (Gy)	Dmax (Gy)	V95% (%)	
1	48	Male	T2N2M0 III	Regional	CTV	66.8	65.0	73.4	100	In-field
2	54	Male	T4N1M0 IV	Local	GTV	65.6	62.1	76.9	100	In-field
3	72	Male	T1N2M0 III	Regional	CTV	67.3	65.1	72.2	100	In-field
4	79	Male	T4N1M0 IV	Local	CTV	64.5	60.6	71.3	100	In-field
5	35	Male	T4N0M0 IV	Local	Marginal to CTV	62.5	55.1	70.3	90.2	marginal
6	29	Male	T2N2M0 III	Local	GTV	70.2	65.7	77.5	100	In-field
7	36	Male	T1N2M0 III	Local	GTV	67.3	61.4	75.8	98.4	In-field
8	32	Female	T2N2M0 III	Regional	CTV	71.2	67.4	78.6	100	In-field
9	35	Male	T4N2M0 IV	Local	CTV	65.5	62.0	74.2	100	In-field
10	41	Female	T1N3M0 IV	Local	GTV	70.5	66.8	78.4	100	In-field
11	52	Male	T3N0M0 III	Local	CTV	63.1	60.7	72.4	100	In-field
12	84	Female	T1N1M0 II	Local	CTV	64.3	60.6	69.4	100	In-field
13	49	Female	T1N2M0 III	Local	CTV	67.8	62.6	74.5	100	In-field
14	70	Female	T4N2M0 IV	Local	Marginal to CTV	66.3	61.9	75.8	100	marginal
15	52	Male	T2N2M0 III	Local	Outside CTV	64.7	59.3	72.0	99.6	outside
16	70	Male	T2N2M0 III	Local	CTV	67.4	63.6	73.3	100	In-field
17	81	Female	T3N2M0 III	Local	CTV	70.6	65.8	78.4	100	In-field
18	55	Male	T2N0M0 II	Local	GTV	64.3	58.8	72.5	100	In-field
